# Preparation and Flame-Retardant Properties of DMMP/Nano-Silica/WPU Composite Materials

**DOI:** 10.3390/polym17081052

**Published:** 2025-04-13

**Authors:** Wanchao Wu, Xiaoyue Huang, Ya Mo, Miaojia Ye, Qian Hu, Quankai Chen, Yiwen Wang, Chuanqun Hu

**Affiliations:** School of Materials and Chemical Engineering, Hubei University of Technology, Wuhan 430068, China; 15972827731@163.com (W.W.); huangxiaoyue0204@163.com (X.H.); moya0102@163.com (Y.M.); 15623677316@163.com (M.Y.); 15827225228@163.com (Q.H.); m13177330069@163.com (Q.C.); 13367232750@163.com (Y.W.)

**Keywords:** waterborne polyurethane, DMMP, nano-silica, flame retardant

## Abstract

Dimethyl methylphosphonate (DMMP) and modified nano-silica were utilised to enhance the mechanical properties, thermal stability, and flame retardancy of waterborne polyurethane (WPU). Nano-silica modified with the silane coupling agent γ-aminopropyltriethoxysilane (KH550) exhibited excellent dispersibility and stability. Compared with pure WPU, the limiting oxygen index (LOI) of P/Si-WPU increased from 18.1% to 28.3%, and its UL-94 rating reached V-0, with a significant improvement in elongation at break. Furthermore, the peak heat release rate of P/Si-WPU decreased by 29.1%, while the total heat release was reduced by 6.8% in comparison to pure WPU. The synergistic flame-retardant mechanism of phosphorus and silicon was investigated through an analysis of the char residue of WPU and its composites. This study provides a potential approach for the development of WPU with superior flame retardancy and enhanced mechanical properties.

## 1. Introduction

With the increasing emphasis on environmental protection and sustainable development, the application of environmentally friendly materials has become more widespread. Waterborne polyurethane (WPU) is a novel eco-friendly material [1], distinguished from traditional polyurethane by its use of water or other polar solvents as a dispersion medium instead of hazardous organic solvents. This substitution not only enhances its physical properties but also reduces odour, minimises volatile organic compound (VOC) emissions, and facilitates modification. Consequently, WPU has found extensive applications in coatings [2], adhesives [3], leather [4], interior decoration materials [5], and the automotive [6] industry. However, WPU exhibits certain limitations, such as inadequate weather resistance, poor thermal stability, and low flame retardancy, which necessitate modification to expand its application scope. In recent years, the rapid advancement of nanotechnology has facilitated the increasing use of nanomaterials, particularly in coatings. Pure WPU has a limiting oxygen index (LOI) of approximately 18%, classifying it as a highly flammable material, which restricts its applicability in specific fields [7]. The incorporation of flame retardants and nano-inorganic materials into WPU has been shown to enhance its thermal stability, flame retardancy, and mechanical strength, thereby broadening its practical applications.

Traditional flame retardants often contain halogen elements, which, while effective in enhancing flame resistance, generate significant amounts of smoke and toxic gases during combustion. This poses substantial risks to human health and the environment, leading to restrictions on their use in many countries. Dimethyl methylphosphonate (DMMP) is an organic phosphorus-based flame retardant that exhibits good compatibility with polymer composites, improving their toughness and plasticity. Due to its high phosphorus content (approximately 25% by mass), DMMP provides effective flame retardancy while producing minimal toxic gas emissions during combustion, making it one of the environmentally friendly flame retardants.

In flame-retardant systems, phosphorus compounds can generate phosphoric acid, metaphosphoric acid, and polyphosphoric acid, which promote the dehydration of polymers to form a char layer. Additionally, phosphorus-containing free radicals can capture combustion-generated radicals, thereby inhibiting further burning [8]. Wang [9] et al. utilised a box-foaming method to prepare flame-retardant polyurethane foam incorporating DMMP, bis(2-hydroxyethyl)amino dimethylphosphonate (BH), and expandable graphite (EG). When the additive concentrations of DMMP, BH, and EG were 8%, 8%, and 6%, respectively, the limiting oxygen index (LOI) of the composite material increased to 30.7%, representing a significant improvement compared to pure polyurethane foam. Furthermore, in cone calorimetry tests, the peak heat release rate of the composite decreased by 40.6%, the total heat release was reduced by 19.0%, and the char residue yield increased by 15.6%.

Nano-silica is an amorphous powder with hydroxyl groups on its surface, produced through the high-temperature hydrolysis of silicon or organosilicon chlorides. It possesses a tetrahedral network structure and, when used as a filler, enhances the fire resistance, water resistance, pollution resistance, and ageing resistance of polymer composites. Consequently, it has been widely applied in the field of polymer composite materials. However, due to its large specific surface area and the abundance of hydroxyl groups on its surface, nano-silica exhibits a hydrophilic polar surface, making it difficult to disperse uniformly in high-viscosity polymer composites, thereby limiting its potential performance. Surface modification with the silane coupling agent KH550 effectively improves this issue. Wang [10] et al. incorporated triazine-derivative-modified nano-silica into a polypropylene matrix alongside an intumescent flame retardant to develop a series of novel intumescent flame-retardant polypropylene composites. The results demonstrated that the limiting oxygen index (LOI) of the composite increased to 29.7%, achieving a UL-94 V-0 rating. Additionally, cone calorimetry tests showed significant reductions in the peak heat release rate (pHRR) and total heat release (THR), indicating a considerable enhancement in flame-retardant performance. Similarly, Zhang [11] et al. studied the synergistic flame-retardant effect of nano-silica and dicyclohexyl hypophosphite in polyamide 66 (PA66). Their findings revealed that when the additive concentrations were 12% and 3%, respectively, the LOI of PA66 increased to 33.1%, with a UL-94 rating of V-0, while both pHRR and THR were significantly reduced in cone calorimetry tests.

Current research suggests that phosphorus and silicon exhibit excellent synergistic flame-retardant effects. In this study, DMMP and nano-silica were combined to compensate for the impact of DMMP on the mechanical properties of WPU, thereby improving its flame-retardant efficiency, thermal stability, and mechanical performance. This enhancement broadens the potential applications of WPU in insulation materials, fire-resistant coatings, and automotive interior components. Furthermore, the synergistic flame-retardant mechanism of the DMMP–nano-silica system was investigated.

## 2. Experimental Section

### 2.1. Raw Materials

The waterborne polyurethane (WPU) was purchased from Anhui Femto Chemical Co., Ltd. (Hefei, China); it is an anionic waterborne polyurethane with a solid content of 42%. Dimethyl methylphosphonate (DMMP) was obtained from Dongguan Xin’an Chemical New Materials Co., Ltd. (Dongguan, China). Nano-silica was sourced from Guangzhou Metal Metallurgy (Group) Co., Ltd. (Guangzhou, China). The silane coupling agent KH550 was supplied by Kangjin New Materials Technology Co., Ltd. (Dongguan, China) Potassium bromide and toluene were procured from Sinopharm Chemical Reagent Co., Ltd. (Shanghai, China). Deionised water was prepared in the laboratory.

### 2.2. Modification of Nano Silicon Dioxide

In a beaker, 50 mL of toluene was used as a solvent, to which 1.5 g of KH550 was added, followed by the addition of 3.5 mL of deionised water and 5.0 g of nano-silica. The mixture was stirred at room temperature using a mechanical stirrer for 2 h. The resulting suspension was then subjected to vacuum filtration, followed by washing with water and drying at 100 °C for 3 h to obtain the modified nano-silica, denoted as K-SiO_2_.

### 2.3. Preparation of DMMP Nano Silicon Dioxide/WPU Composite Materials

The waterborne polyurethane (WPU) composite material was prepared using a physical–mechanical blending method. An appropriate amount of WPU emulsion was placed in a beaker, followed by the addition of a specified quantity of DMMP and modified nano-silica. The mixture was stirred at room temperature using a magnetic stirrer for 4 h, then left to stand for 30 min to eliminate air bubbles. The resulting composite solution was then coated onto the surface of a polytetrafluoroethylene (PTFE) mould. After drying at room temperature for 8 h, the material was placed in a vacuum drying oven and heated at 60 °C for 12 h to obtain the modified WPU composite. The mass fraction of the flame retardant in the composite material was 9%.

### 2.4. Measurement and Characterisation

#### 2.4.1. Physical Properties of PU Composite Materials

A series of physical properties of the WPU composite materials were evaluated, including appearance, storage stability, average emulsion particle size, and zeta potential.

The storage stability of the WPU composite emulsion is a critical factor in determining its shelf life. The specific testing method was as follows: equal amounts of the WPU composite emulsion were symmetrically placed in a centrifuge and subjected to centrifugation at 3000 r/min for 15 min. If no sedimentation was observed, the emulsion was considered stable for storage over a period of six months.

#### 2.4.2. Structural Characterisation

Fourier transform infrared (FT-IR) spectroscopy was used to analyse the thin films of different composite materials, with a scanning range of 400~4000 cm^−1^.

#### 2.4.3. Mechanical Properties

According to the national standard GB/T 1040.3-2006, the composite material was fabricated into dumbbell-shaped specimens. The thickness and width of each specimen were measured using a vernier calliper. The tensile strength of the specimens was then tested using a universal testing machine at a tensile rate of 100 mm/min, with multiple measurements taken to determine the average value.

Hardness testing was conducted in accordance with GB/T 531-2008 using a Shore A durometer. A sample with a thickness of no less than 6 mm was placed on a horizontal platform, and the durometer’s indenter was pressed into the composite material. The test was repeated five times, and the average value was recorded.

#### 2.4.4. Thermal Analysis

Thermogravimetric analysis (TGA) was performed using a thermogravimetric analyser. Approximately 10 mg of each sample was placed in a small crucible and heated to 600 °C under a nitrogen atmosphere at a heating rate of 10 °C/min. The relevant thermal stability parameters were obtained, including the initial decomposition temperature (T_5%_), the temperature at 50% weight loss (T_50%_), and the char residue at 600 °C (R_600_).

The thermal conductivity and thermal diffusivity of WPU and its composites were evaluated using a laser flash analyser.

#### 2.4.5. Flammability Testing

The flammability of polymer plastics was characterised using three key tests: the limiting oxygen index (LOI) test, the vertical burning (UL-94) test, and the cone calorimetry (CCT) test.

The LOI test was conducted in accordance with GB/T 2406.2-2009. Samples were prepared with dimensions of 140 × 50 × 2 mm (length × width × thickness). The top surface ignition method was used, in which the innermost part of the flame was directed at the top surface of the sample for 30 s, with the flame being removed every 5 s. If the ignition time exceeded 180 s or the burned length exceeded 50 mm, the oxygen concentration was deemed insufficient to achieve self-extinguishment, and the test was repeated with a lower oxygen concentration. Each sample was tested at least five times to ensure accuracy.

The UL-94 vertical burning test was performed according to GB/T 2408-2021. Samples were prepared with dimensions of 130 × 13 × 1.5 mm. The sample was positioned approximately 300 mm above a cotton pad, and a blowtorch was used to ignite the sample 10 mm below its centre at a 45° angle to the horizontal plane. Ignition was performed twice, each for 10 s, while observing the burning behaviour and the presence of molten drips. Each set of samples was tested five times.

The cone calorimetry test (CCT) was conducted at a heat flux of 35 kW, simulating the burning behaviour of the samples under real fire conditions. The initial temperature was set to 25 °C, and key parameters such as heat release rate (HRR), total heat release (THR), smoke production rate (SPR), and total smoke production (TSP) were measured.

#### 2.4.6. Analysis of Residual Carbon

A muffle furnace was used to heat the WPU films, both before and after modification, to induce thermal decomposition. The heating temperature was set to 400 °C, and the heating duration was 10 min, resulting in the formation of char residues from WPU and its composites. The char residues were characterised using scanning electron microscopy (SEM), X-ray photoelectron spectroscopy (XPS), and Raman spectroscopy to analyse their structure and investigate the condensed-phase flame-retardant mechanism of the composite materials.

## 3. Results and Discussion

### 3.1. Modification of Nano Silicon Dioxide

**Figure 1:**
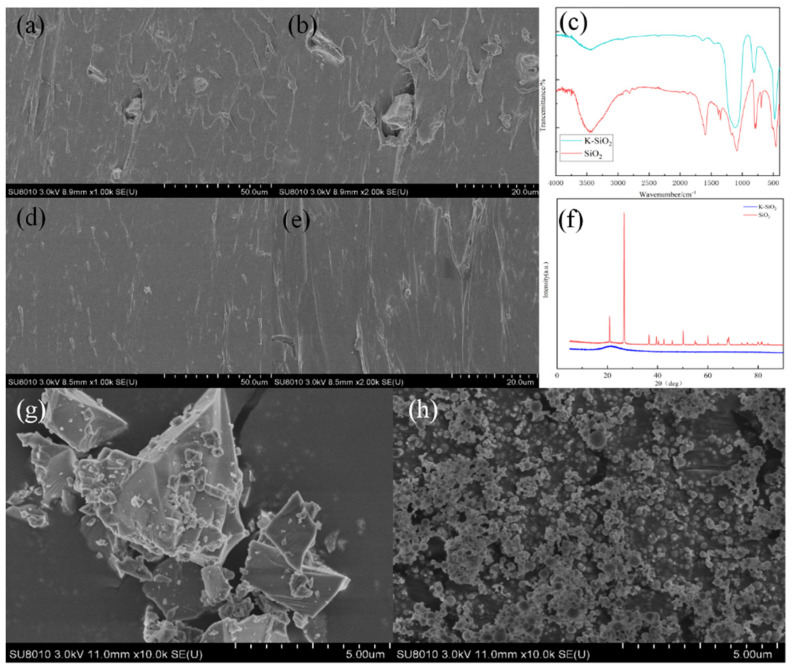
SEM images of the dispersion state of nano-silica in WPU before and after modification: (**a**,**b**) before modification; (**d**,**e**) after modification; (**c**) FT-IR of nano-silica before and after modification; (**g**) SEM images of nano-silica before and after (**h**) modification; (**f**) XRD of nano-silica before and after modification.

To enhance the dispersion of nano-silica in waterborne polyurethane (WPU), its surface was modified using KH-550. The modification process involved the dehydration condensation reaction between KH-550 and hydroxyl groups on the SiO_2_ surface, reducing the hydroxyl concentration and thereby improving the dispersion of nano-SiO_2_. As shown in Figure 1c, the stretching vibration peak of Si-O-Si appears around 1100 cm^−1^, while the stretching vibration peak of -OH is observed near 3400 cm^−1^. After modification, the intensity of the -OH stretching vibration peak decreases significantly, indicating the successful modification of nano-SiO_2_.

The dispersion state of nano-silica in WPU significantly affects its mechanical properties. As depicted in Figure 1a,b, nano-silica exhibits agglomeration in WPU, leading to defects in the fracture surface. This is attributed to the high concentration of hydroxyl groups on the nano-SiO_2_ surface, which tend to form hydrogen bonds and aggregate, making uniform dispersion difficult in the high-viscosity polymer matrix. Consequently, the interfacial adhesion between nano-silica and the polymer matrix is weak, adversely affecting the mechanical properties of the composite material. In contrast, Figure 1d,e demonstrate a more uniform and orderly dispersion of nano-silica, with no visible agglomeration. The modified nano-silica exhibits improved compatibility with the polymer matrix, contributing to enhanced mechanical performance of the composite material.

As shown in Figure 1f, the modified nano-silica exhibits a single broad and diffuse peak, indicating its transformation from a crystalline to an amorphous state. This conclusion is further supported by Figure 1g,h, which confirm the amorphous nature of the modified nano-silica. Amorphous nano-silica possesses a larger specific surface area, which enhances its interaction with the polymer matrix. The incorporation of amorphous nano-silica into the composite material can improve its mechanical strength. Additionally, during the condensed-phase flame-retardant process, the presence of amorphous nano-silica enhances the integrity of the carbon–silica layer, effectively preventing the transfer of heat and combustible substances during combustion.

### 3.2. Stability and Hydrophobicity of WPU Lotion and Its Composites

**Figure 2:**
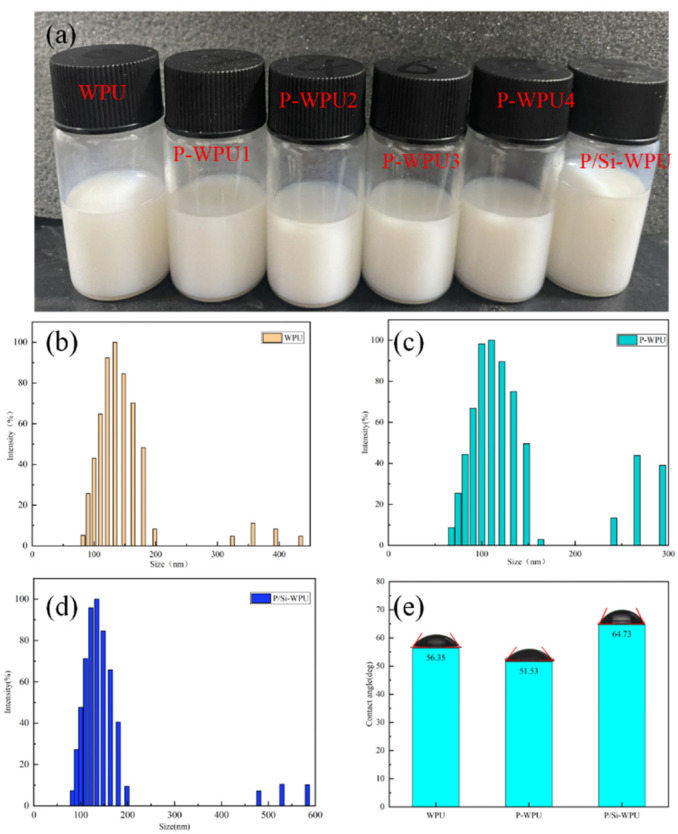
(**a**) Appearance of WPU and its composite materials; (**b**–**d**) particle size of WPU and its composite materials; (**e**) contact angle of WPU and its composite materials. (The added amounts of DMMP from left to right in (**a**) are 0.2%, 4%, 6%, and 8%, respectively. The added amounts of DMMP and nano-SiO_2_ in P/Si-WPU are 8% and 1%, respectively; the same below).

A series of waterborne polyurethane (WPU) samples were characterised based on their appearance, storage stability, particle size distribution, zeta potential, and contact angle to evaluate their fundamental physical properties. As shown in Figure 2a and Table 1, all WPU samples exhibited a milky white colour with a bluish tint. The pure WPU displayed the most pronounced blue hue, indicating the smallest particle size in the absence of flame retardants. After subjecting the WPU emulsions to centrifugation, no sedimentation was observed, confirming their excellent storage stability with a shelf life exceeding six months. Furthermore, the absolute values of the zeta potential for WPU, P-WPU, and P/Si-WPU emulsions were all greater than 30 eV. According to colloidal stability principles, this suggests that these WPU emulsions possess excellent dispersibility and long-term storage stability [12].

The stability of the emulsion mainly depends on its particle size distribution [13]. It can be seen from Figure 2b–d and Table 1 that the effective particle size of WPU without flame retardant is 128.78 nm. After adding 8% DMMP, the particle size has little effect, increasing to 129.47 nm. This is because DMMP is a liquid flame retardant and can be evenly dispersed in WPU. After adding nano-silica, the particle size of the emulsion has a more obvious increase, increasing to 141.86 nm. It can also be seen from the particle size distribution diagram that large particle aggregates appear in the P/Si-WPU emulsion. The reason for this phenomenon is that the modified nano-silica still has a very high specific surface energy and a small volume effect. The high specific surface energy makes K-SiO_2_ in an energy unstable state and easy to agglomerate. In addition, the small amount of -OH carried on its surface can also cause dehydration condensation between particles to form agglomerates with hydrogen bonds and chemical bonds, thereby increasing the effective particle size of the emulsion. The hydrophilicity of WPU and its composite films was characterised using a contact angle measuring instrument, and the results are presented in Table 1 and Figure 1e. The unmodified WPU film exhibited typical hydrophilic properties, with an average contact angle of 56.35°. Upon the addition of a certain amount of DMMP, the contact angle of P-WPU decreased to 51.53°, indicating a slight increase in hydrophilicity due to the intrinsic hydrophilic nature of phosphorus. In contrast, the incorporation of nano-silica led to an increase in the contact angle of the WPU composite film to 64.73°. This can be attributed to the presence of a substantial number of Si–O–Si bonds in the K-SiO_2_ system. Since siloxane bonds exhibit low surface energy, they contribute to enhanced hydrophobicity, thereby improving the water resistance of the composite material.

### 3.3. Mechanical Properties and Thermal Analysis of WPU and Its Composite Materials

**Figure 3:**
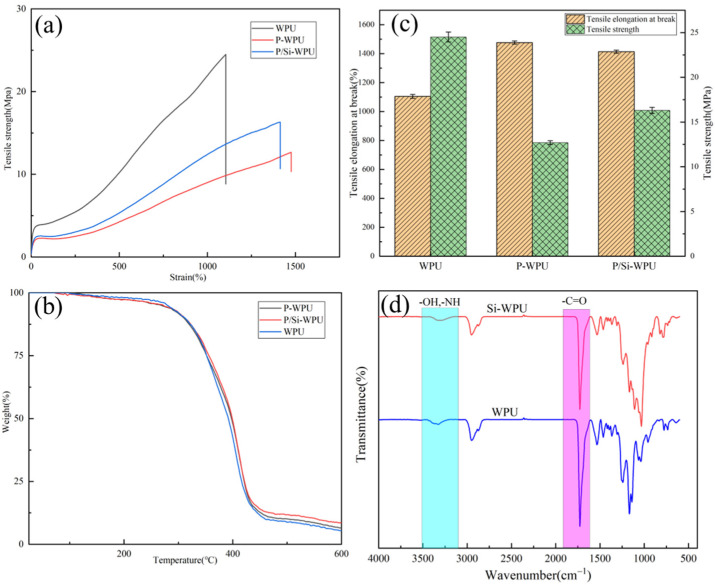
(**a**,**c**) Tensile strength and elongation at break of WPU and its composite materials; (**b**) WPU and its composite material TG; (**d**) infrared spectra of WPU and its composites.

Mechanical performance is an important factor affecting the usage scenarios of flame-retardant WPU; therefore, it is necessary to conduct mechanical performance testing on WPU and its composite materials. Table 2 and Table 3 and Figure 3 all show a series of performance indicators of WPU and its composite materials.

From Table 2 and Figure 3a,c, it can be seen that with the addition of DMMP and nano-SiO_2_, the fracture elongation of the composite material increases. Compared with pure WPU, the fracture elongation of P-WPU and P/Si WPU increases from 1104.8% to 1476.1% and 1413.1%, respectively. However, there was a significant decrease in tensile strength and Shore hardness in the material with only DMMP added, which was improved after the addition of nano-SiO_2_.

The thermal properties of WPU and its composites were analysed using thermogravimetric analysis (TGA) and laser flash diffusivity measurements, with the results presented in Figure 3b and Table 3. The incorporation of DMMP led to an earlier thermal decomposition of WPU. This phenomenon occurs because DMMP decomposes in the early stages of combustion, promoting polymer dehydration and resulting in a reduction in composite mass [14]. However, with the addition of nano-SiO_2_, the initial decomposition temperature increased. This can be attributed to the presence of hydroxyl (-OH) groups on the surface of K-SiO_2_, which interact with amide or ester carbonyl groups in WPU via hydrogen bonding. From Figure 3d, it can be observed that the addition of nano-silica results in a shift in the –OH stretching vibration peak towards a lower wavenumber, accompanied by a reduction in the intensity of the C=O stretching vibration peak. This phenomenon suggests, to some extent, that –OH interacts with the C=O groups in WPU through hydrogen bonding [15]. This interaction enhances molecular entanglement, increasing intermolecular forces and ultimately improving the thermal stability of the composite. Furthermore, the residual mass at 600 °C increased with the incorporation of DMMP and nano-SiO_2_. Additionally, a decrease in the thermal conductivity of the composites was observed with the addition of nano-SiO_2_. This can be attributed to the structural transformation of modified nano-SiO_2_ from a crystalline to an amorphous form, altering the heat transfer mechanism. Unlike in a crystalline structure where heat propagates in wave-like forms, heat diffusion in the polymer occurs more slowly [16]. These findings indicate that the incorporation of DMMP and nano-SiO_2_ into WPU can effectively enhance the thermal stability of the composite material.

### 3.4. Flame Retardant Performance Analysis

The flame-retardant properties of WPU and its composite materials were characterised using the limiting oxygen index (LOI), vertical burning test (UL-94), and cone calorimetry (CONE). LOI and UL-94 primarily assess the self-extinguishing capability and combustion characteristics of WPU and its composites, while cone calorimetry simulates the combustion behaviour of polymers under real fire conditions [17].

The LOI and UL-94 test results are presented in Table 4. The LOI of pure WPU is only 18.1%, classifying it as a highly flammable organic polymer. In comparison, the LOI values of P-WPU and P/Si-WPU increased to 27.6% and 28.3%, respectively, reaching the classification of flame-retardant polymers. In the UL-94 test, pure WPU exhibited significant molten dripping within 10 s of ignition, and the dripping material ignited the underlying cotton pad, resulting in no UL-94 rating. In contrast, both P-WPU and P/Si-WPU produced only a small number of molten drips, which did not ignite the cotton pad, and the flame self-extinguished upon removal of the ignition source, achieving a UL-94 V-0 rating. The LOI and UL-94 results demonstrate that phosphorus and silicon elements exhibit a synergistic effect in the flame-retardant process, effectively enhancing the fire resistance of the material.

**Figure 4:**
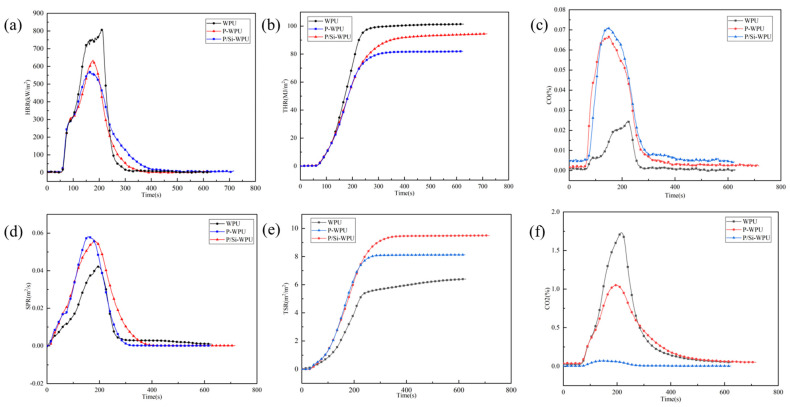
Cone calorimetry results of WPU and its composite materials. (**a**) HRR; (**b**) THR; (**c**) CO; (**d**) SPR; (**e**) TSR; (**f**) CO_2_.

The cone calorimetry data for WPU and its composite materials are presented in Figure 4, with detailed results listed in Table 5. The time to ignition (TTI) for pure WPU is 53 s, while the TTI of P-WPU and P/Si-WPU decreases to 45 and 48 s, respectively. The earlier ignition time observed with the incorporation of flame retardants is attributed to the phosphorus-based flame-retardant DMMP, which promotes dehydration and char formation, thereby accelerating polymer thermal decomposition. During combustion, the peak heat release rate (pHRR) and total heat release (THR) of pure WPU are 809.37 kW/m^2^ and 101.39 MJ/m^2^, respectively. After introducing flame retardants, the pHRR and THR of P/Si-WPU decrease to 574.21 kW/m^2^ and 94.47 MJ/m^2^, respectively. This reduction indicates that P/Si-WPU exerts a lower thermal impact on the surrounding environment during combustion, contributing to improved fire safety.

The fire growth index (FGI) is introduced to evaluate the rate of flame propagation per unit time, allowing for a better prediction of fire spread trends [18]. The FGI of pure WPU is 3.87, whereas that of P/Si-WPU decreases to 3.54, demonstrating that the addition of flame retardants reduces the flame propagation capability, thereby enhancing fire control in emergency situations. These findings confirm that the incorporation of DMMP and nano-silica significantly enhances the fire safety performance of the composite material.

The smoke production rate (SPR) and total smoke release rate (TSR) are key factors in understanding the combustion characteristics of polymers during fires, as shown in Figure 4d,e. The results showed that the SPR peak of pure WPU was 0.057 m^2^/s, while the SPR peaks of P-WPU and P/Si WPU were 0.040 m^2^/s and 0.066 m^2^/s, respectively. This is because the addition of flame retardants promotes the conversion of polymers to hydrocarbons, which leads to a decrease in CO_2_ yield. Therefore, more substances are converted into smoke rather than gas during combustion. The production of CO_2_ usually means complete combustion of the polymer [19]. As can be seen from Figure 4c,f, the addition of flame retardants significantly reduces the amount of CO_2_ generated, indicating that the addition of DMMP and nano-SiO_2_ inhibits the complete combustion of WPU.

### 3.5. Residual Carbon Analysis

**Figure 5:**
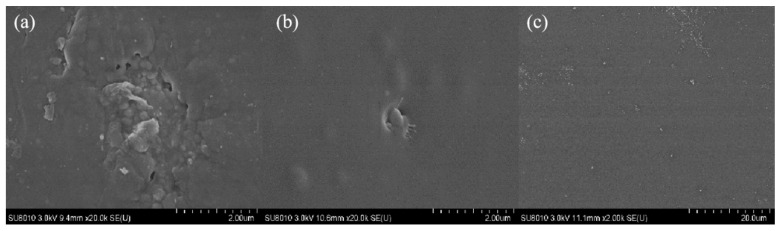
SEM image of residual carbon from WPU and its composite materials.

In order to further reveal the flame-retardant mechanism of WPU and its composite materials, SEM was used to characterise the condensed-phase products of WPU, P-WPU, and P/Si WPU after combustion, as shown in Figure 5a. It can be clearly seen from the SEM image of the residual carbon that pure WPU has many pores on the outer surface of the residual carbon, and the entire carbon layer is also relatively loose. This phenomenon indicates that the residual carbon after pure WPU combustion cannot form a good flame-retardant layer, and these pores can cause combustible gases and heat generated during combustion to enter the interior of the material, promoting violent combustion of the material. With the introduction of DMMP, the residual carbon surface of P-WPU becomes denser and the pores decrease, indicating an improvement in its ability to block oxygen, heat, and other flammable gases, as shown in Figure 5b. From Figure 5c, it can be seen that the carbon layer of P/Si WPU is more continuous and dense. This structure can successfully prevent heat transfer and combustible gas exchange, and can also prevent volatile gases generated by combustion from escaping from the interior of the material. A conclusion can be drawn from the above phenomenon: the addition of organic phosphorus flame retardants and nano-silica can significantly change the structure and morphology of residual carbon in composite materials, thereby forming an effective barrier on the surface to prevent further combustion of internal materials.

**Figure 6:**
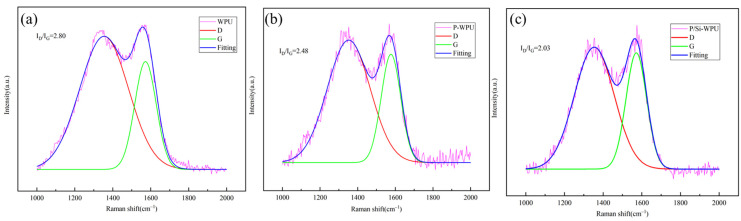
Raman spectra of WPU and its composite materials.

**Figure 7:**
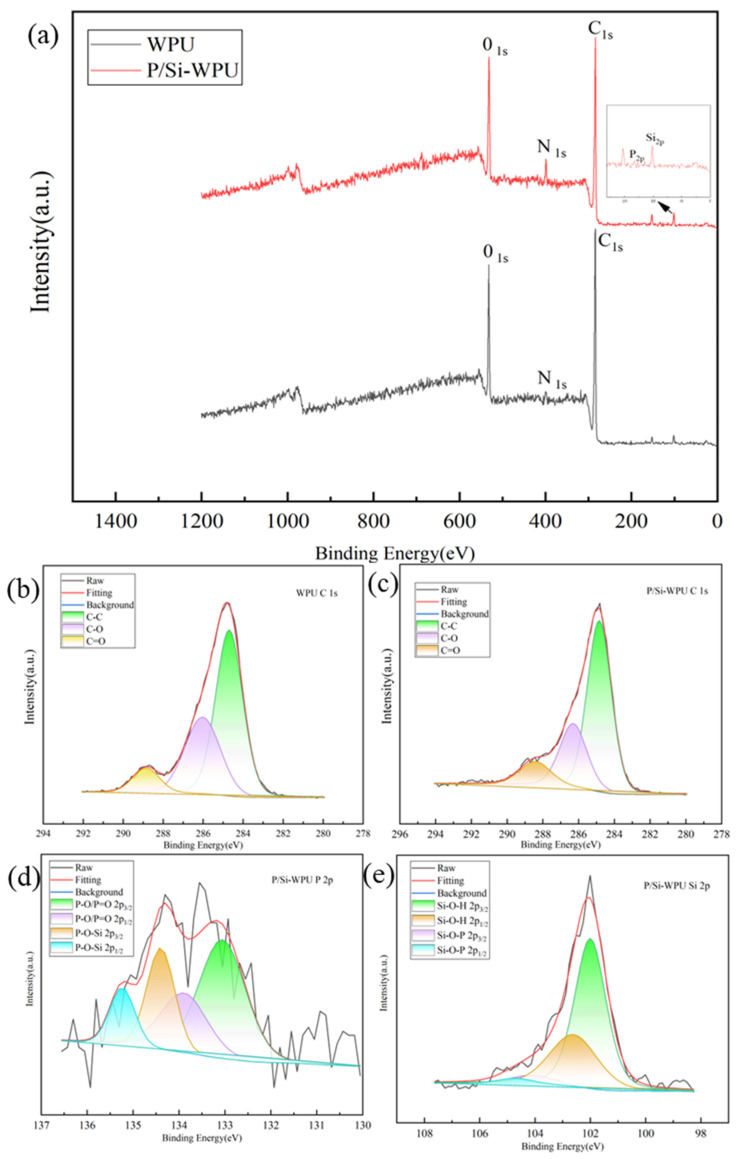
(**a**) XPS spectra of WPU and its composite material coke; (**b**,**c**) high-resolution map of C1s and (**d**) high-resolution map of P2p; (**e**) high-resolution map of Si2p.

To further investigate the flame-retardant mechanism of the char layer, Raman spectroscopy was employed to determine the graphitisation degree of the residual char in WPU and its composites. The Raman spectra exhibit two distinct characteristic peaks at approximately 1350 cm^−1^ and 1580 cm^−1^, corresponding to the D-band and G-band, respectively. The D-band primarily originates from the stretching vibrations of C–C and C=C bonds, with its intensity and shape reflecting the structural characteristics and defect levels of the sample. The G-band is associated with the stretching vibrations of sp^2^ hybridised carbon atoms and π electrons, providing insights into the layering and topological structure of the material. The degree of graphitisation in the residual char is negatively correlated with the ID/IG ratio [20]. As shown in Figure 6a, the ID/IG ratio for pure WPU is 2.80, as shown in Figure 6b,c, the ID/IG ratios for P-WPU and P/Si-WPU are 2.48 and 2.03, respectively. This result shows that the P/Si-WPU composites have the highest degree of residual carbonization. The enhanced graphite structure indicates that the carbon layer formed after the combustion of P/Si-WPU is denser and smoother, providing more effective thermal insulation and preventing the release of flammable gases. This conclusion is consistent with the observations of SEM images, which further confirms the superior blocking effect of the P/Si-WPU composite carbon layer.X-ray photoelectron spectroscopy (XPS) was employed to analyse the elemental composition and chemical bonding states on the surface of the composite materials, and the results are presented in Figure 7. The XPS survey spectrum of P/Si-WPU reveals the appearance of two new characteristic peaks at approximately 101.1 eV and 133 eV, corresponding to Si 2p and P 2p, respectively. This confirms that phosphorus and silicon actively participate in the formation of the char layer during the flame-retardant process of the composite material.

As shown in Figure 7c, the elemental composition of the residual char in P/Si-WPU consists of 70.28% carbon, 20.05% oxygen, 6.72% nitrogen, 2.20% silicon, and 0.74% phosphorus. The C1s spectra of both WPU and P/Si-WPU exhibit characteristic peaks at 284.84 eV (aromatic C–C and C–H bonds), 286.30 eV (C–OH, C–O–P, or C–N bonds), and 288.41 eV (C=O bonds). Semi-quantitative analysis, as summarised in Table 6, indicates that P/Si-WPU retains a higher proportion of oxygen-bound C=O bonds, which possess stronger binding energy, thereby enhancing the stability of the residual char.

In the P2p spectrum of P/Si-WPU, the peaks at 132.97 eV and 133.81 eV correspond to P–O and P=O bonds in the 2p_3_/_2_ and 2p_1_/_2_ states, respectively. These species are primarily attributed to the thermal decomposition of DMMP, which generates phosphoric acid and polyphosphoric acid. Additionally, the peaks at 134.33 eV and 135.17 eV correspond to P–O–Si in the 2p_3_/_2_ and 2p_1_/_2_ states, respectively. The Si2p spectrum of P/Si-WPU further reveals two distinct chemical states, Si–O–H and Si–O–P. Notably, the Si–O–P bond exhibits high thermal stability, which can delay the thermal decomposition of the composite material while promoting the char formation process facilitated by DMMP.

These findings provide strong evidence that phosphorus and silicon exhibit a synergistic effect in the condensed-phase flame-retardant mechanism, contributing to enhanced fire resistance of the composite material.

### 3.6. Flame-Retardant Mechanism Analysis

**Figure 8:**
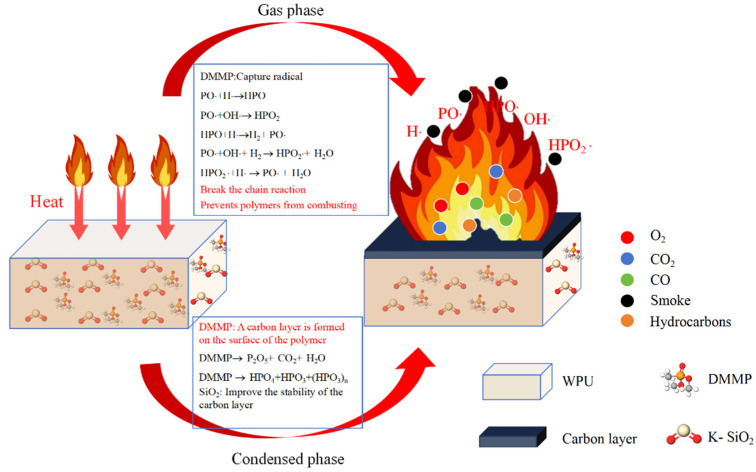
Schematic diagram of flame retardancy of composite materials.

The flame-retardant process of composite materials was investigated using TG, Raman, XPS, and SEM. From the above experimental results, it can be found that with the addition of flame-retardant components, the formation of surface carbon layer in composite materials will be accelerated during combustion, thereby slowing down the combustion rate. The possible combustion mechanism of flame-retardant WPU is shown in Figure 8. The flame-retardant process is divided into gas-phase and condensed-phase flame retardants. Based on previous literature reports [15,21,22], in gas-phase flame retardants, the phosphorus-containing flame retardant will generate PO· after heating to capture the H· and OH· generated during the combustion process, thereby inhibiting the generation of free radicals and interrupting the chain reaction. In the condensed phase, DMMP generates phosphoric acid, pyrophosphate, and polyphosphoric acid upon heating to promote the dehydration of the polymer and form a carbon layer. A protective layer is formed on the surface of the polymer to prevent the transfer of heat and combustible gases. However, the presence of pores on the surface of the carbon layer with only DMMP indicates that its density still needs to be improved. After adding nano-SiO_2_, the carbon layer can have a higher degree of graphitization, which is conducive to the formation of a denser carbon layer. In summary, the synergistic effect of P and Si can better improve the flame-retardant performance of WPU.

## 4. Conclusions

In summary, a novel phosphorus- and silicon-containing flame-retardant system for waterborne polyurethane (WPU) was developed by incorporating nano-SiO_2_ and DMMP. Structural characterisation using SEM, XRD, and FT-IR confirmed the successful modification of nano-SiO_2_, which exhibited excellent dispersibility and stability. Notably, even at a low addition level, the modified nano-SiO_2_ significantly enhanced both the flame retardancy and mechanical properties of the composite material. Compared with pure WPU, the LOI of P/Si-WPU increased from 18.1% to 28.3%, classifying it as a flame-resistant material, while its UL-94 rating improved to V-0. Cone calorimetry tests further demonstrated that the incorporation of organic phosphorus flame retardants and nano-SiO_2_ resulted in a 29.1% reduction in peak heat release rate (pHRR) and a 6.8% decrease in total heat release (THR). Additionally, the elongation at break of P/Si-WPU was significantly improved compared with pure WPU.

The gas-phase and condensed-phase flame-retardant mechanisms of WPU and its composites were investigated by analysing the char residues. The synergistic effect of DMMP and nano-SiO_2_ promoted polymer dehydration during combustion, leading to the formation of a protective char layer, which effectively shielded heat and combustible gases, thereby achieving enhanced flame retardancy.

## Figures and Tables

**Table 1:** Physical properties of WPU and its composite materials.

Characteristic	WPU	P-WPU	P/Si-WPU
Colour	milky white	milky white	milky white
Zeta potential (mV)	−66.52	−65.62	−61.10
Effective particle size (nm)	128.78	129.47	141.86
Stability	≥6 months	≥6 months	≥6 months
Contact angle	56.35	51.53	64.73

**Table 2:** Mechanical properties of WPU and its composite materials.

Physical Characteristics	WPU	P-WPU	P/Si-WPU
Tensile strength (Mpa)	24.5 ± 0.55	12.7 ± 0.21	16.3 ± 0.34
Tensile elongation at break (%)	1104.8 ± 13.2	1476.1 ± 11.3	1413.1 ± 10.6
Shore hardness (HA)	93.0 ± 1.1	72.0 ± 2.3	87.0 ± 1.8

**Table 3:** Thermal analysis data of WPU and its composite materials.

	WPU	P-WPU	P/Si-WPU
*T*_5%_ (°C)	279.8	268.9	272.8
*T*_10%_ (°C)	311.9	310.2	312.1
*T*_50%_ (°C)	391.9	397.6	405.1
*R*_600_ (%)	5.5	6.4	8.7
Thermal diffusivity coefficient (mm^2^/s)	0.209	0.099	0.087
Thermal conductivity (W/(m·K))	0.385	0.230	0.206

**Table 4:** LOI and UL-94 test data of WPU and its composite materials.

Sample	LOL(%)	UL-94 Test		
		UL-94 Rating	Dripping	Ignite the Cotton
WPU	18.1 ± 0.2	No rating	Yes	Yes
P-WPU	27.6 ± 0.1	V-0	Yes	No
P-WPU1	21.6 ± 0.3	No rating	Yes	Yes
P-WPU2	23.0 ± 0.1	No rating	Yes	Yes
P-WPU3	25.2 ± 0.2	V-1	Yes	No
P/Si-WPU	28.3 ± 0.3	V-0	Yes	No

Note: The addition of DMMP in P-WPU1, P-WPU2, and P-WPU3 were 2%, 4%, and 6%, respectively.

**Table 5:** Cone calorimetry data of WPU and its composite materials.

Samples	TTI (s)	THR (MJ/m^2^)	PHRR (kW/m^2^)	TSR (m^2^/m^2^)	FGI
WPU	53.0	101.39	809.37	6.40	3.87
P-WPU	45.0	81.98	636.55	8.12	3.61
P/Si-WPU	48.0	94.47	574.21	9.50	3.54

**Table 6:** XPS determination results of residual carbon C 1s for WPU and P/Si WPU.

Sample	C-C	C-O	C=O
WPU	61.89%	31.43%	6.68%
P/Si-WPU	61.36%	27.06%	11.58%

## Data Availability

The original contributions presented in this study are included in the article. Further inquiries can be directed to the corresponding author.
